# Effects of Antifibrotic Therapy in Patients with Combined Pulmonary Fibrosis and Emphysema: A US-Based Cohort Study

**DOI:** 10.3390/biomedicines13102522

**Published:** 2025-10-16

**Authors:** Abhishek Shah, Esteban Kosak Lopez, Andrew Geller, Maanav Patel, Sadia Benzaquen

**Affiliations:** 1 Department of Medicine, Jefferson Einstein Philadelphia Hospital, Philadelphia, PA 19141, USA; esteban.kosaklopez@jefferson.edu (E.K.L.);; 2 Philadelphia College of Osteopathic Medicine, Philadelphia, PA 19131, USA; 3 Division of Pulmonary and Critical Care Medicine, Jefferson Einstein Philadelphia Hospital, Philadelphia, PA 19141, USA

**Keywords:** lung diseases, interstitial, pulmonary fibrosis, emphysema

## Abstract

**Background/Objectives**: Combined pulmonary fibrosis and emphysema (CPFE) is associated with poor outcomes. We investigated the association of antifibrotic therapy on patients with CPFE. **Methods**: This retrospective study included adult patients, older than 18 years, with a diagnosis of CPFE between 2015 and 2019 using TrinetX database. CPFE was defined as a diagnosis of pulmonary fibrosis (PF) and emphysema or chronic obstructive pulmonary disease. Propensity score matching was performed to compare baseline characteristics for CPFE patients on antifibrotic therapy (nintendanib and pirfenidone) with those not on antifibrotic therapy. The outcomes studied included all-cause mortality, major adverse cardiac event (MACE, [myocardial infarction, unstable angina]), hypoxic and hypercapnic respiratory failure, and stroke. These outcomes were compared at one-, three-, and five-year follow-ups. **Results**: Patients were divided into two cohorts: those on antifibrotic therapy (cohort 1, n = 861) and those without antifibrotic therapy (cohort 2, n = 861). Although not statistically significant, there was a trend towards increased mortality in cohort 1 at the 5-year follow-up (HR 1.14; CI 0.99–1.33). There was also an increased incidence of MI (HR 1.68; CI 0.88–1.47) and hypoxic respiratory failure (HR 1.17; CI 0.99–1.39). Notably, there was also a trend towards decreased incidence of stroke (HR 0.73; CI 0.51–1.05), and no difference in unstable angina (HR 0.94; CI 0.47–1.86) and hypercapnic respiratory failure (HR 0.99; CI 0.67–1.47). **Conclusions**: For patients with CFPE, antifibrotic use demonstrated a trend towards increased risk of mortality at 5-year follow-up, raising concerns for “sicker patient” bias. Prospective studies should be designed to include patients with CPFE and evaluate the benefits of antifibrotics.

## 1. Introduction

Chronic pulmonary fibrosis and emphysema (CPFE) is a severe disorder, in which patients have features of both pulmonary fibrosis (PF) and emphysema. This syndrome is relatively new, as it was first described in 2005 [1]. The current diagnosis is complex, involving a combination of risk factors, physiological testing, and radiographic imaging. Prevalent risk factors include cigarette smoking history, predominantly male gender, and inhalational exposures [2].

Diagnostic testing for CPFE includes physiological testing with spirometry and radiographic imaging with high-resolution CT scans (HRCT). Spirometry can show relatively preserved lung volumes, such as vital capacity and total lung capacity, along with a relatively normal ratio for expiratory volume in one second (FEV1) to forced vital capacity (FVC). A proposed mechanism for this phenomenon can be attributed to the opposing pathophysiologic effects of emphysema and PF. However, these patients will likely have decreased diffusing capacity of the lungs for carbon monoxide, indicating that gas exchange has been impaired [3] Radiographically, there should be findings of emphysema (mostly upper lobe-predominant) and usual interstitial pattern (mostly lower lobe-predominant) in the HRCT [1].

CPFE has been associated with increased mortality when compared to emphysema or PF alone [4,5]. Median survival for these patients was as high as 8.5 years. However, survival rates dropped to as low as 60% at 1-year follow-up in particular subgroups, such as CPFE patients who also had pulmonary hypertension [6]. Lung cancer was another observed common complication, with the most common types being squamous cell carcinoma and adenocarcinoma [7].

Although the current evidence has demonstrated the rising prevalence of CPFE, the role of pharmacologic therapy has been poorly understood [8]. Treatment is primarily based on supportive therapy, such as smoking cessation, oxygen supplementation, and pulmonary rehabilitation [8]. Antifibrotics such as nintedanib and pirfenidone have been considered, given that they have shown survival benefits in patients with isolated PF [9,10]. This study aims to examine whether antifibrotic therapy has a role in the treatment of patients with CPFE. Examining the effects of antifibrotic therapy on CPFE can help to better understand the disorder and serve as a potential first step in developing a true pharmacologic treatment.

## 2. Materials and Methods

We conducted a retrospective cohort analysis from 1 January 2015, to 31 December 2019, using the TriNetX database on 13 April 2025. TriNetX is a global database that includes real-time electronic medical records from 67 US-based healthcare organizations and has been approved by the Western Institutional Review Board, as only de-identified data is used. TrinetX is compliant with all regulatory requirements and does not require separate IRB submission or approval from our local institution’s IRB (Jefferson Health IRB). Since TrinetX is a platform that provides de-identified, aggregated, and standardized electronic health record (EHR) data, it is generally not considered “human subjects research” and is therefore exempt from the requirement for institutional IRB oversight at the individual researcher level. All analyses were performed in the TriNetX “Analytics” network using real-time analytics features. The information about how to implement TrinetX for research has been described elsewhere [11].

**Figure 1:**
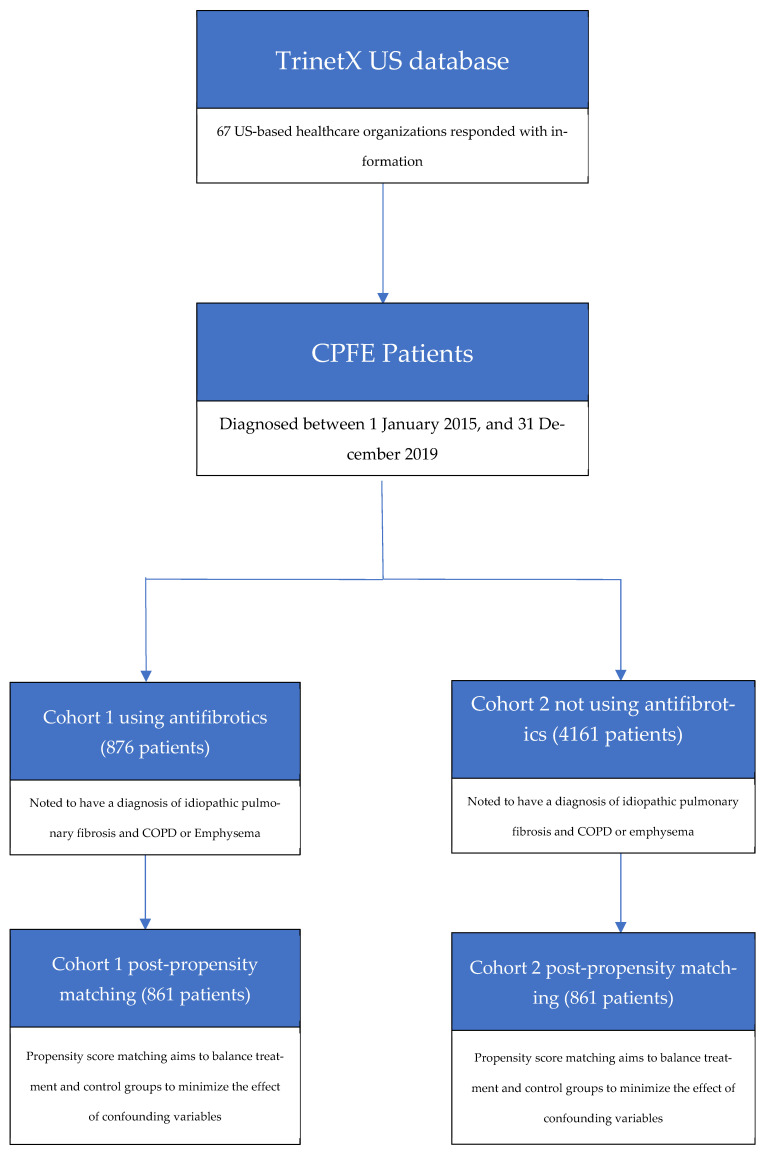
Cohort creation flowchart.

Patients with ages older than 18 years and a diagnosis of CPFE were identified through ICD-10 codes. CPFE was defined in patients having a diagnosis of pulmonary fibrosis (ICD10CM:J84.112) and emphysema (ICD10CM:J43) or chronic obstructive pulmonary disease (ICD10CM:J44.9). Then, two cohorts were created, in which cohort 1 included patients with antifibrotic use (including nintedanib and pirfenidone) and cohort 2 was patients with no antifibrotic use. Figure 1 describes the workflow process for the cohort creation.

Analysis of outcomes was calculated at 1-year, 3-year, and 5-year follow-ups after the index event, which was defined as the first day of meeting all criteria, including diagnosis of CPFE and starting antifibrotic therapy for cohort 1, and diagnosis of CPFE only for cohort 2. The primary outcome studied was the overall impact of antifibrotic use on all-cause mortality in patients with CPFE. Secondary outcomes included the incidence of major adverse cardiac events [MACE] (ICD10CM:I21.9, ICD10CM:I20.0), stroke (ICD10CM:I63), and acute hypoxic and hypercapnic respiratory failure (ICD10CM:J96.01, ICD10CM:J96.02) in patients taking antifibrotics.

Since this study was conducted as a retrospective cohort analysis, the statistical power was not performed. Instead, both cohorts were balanced using propensity score matching, which was performed using a multivariable logistic regression accounting for demographics (age at index, race, gender, and body mass index), comorbidities (respiratory, cardiovascular, and metabolic status; neoplasms; smoking status; and autoimmune diseases), laboratory results (hemoglobin A1c, brain natriuretic peptide, and C-reactive protein), and medications (immunosuppressants, biologics, systemic corticosteroids, inhaled therapies, antidiabetics, anti-hypertensives, diuretics, and anti-lipemics), as noted in Table 1. This process of propensity score matching resulted in 861 patients in both cohorts, which was appropriate for a rare disorder such as CPFE.

The exposure for outcomes was at 1 year, 3 years, and 5 years after the index event during the time window. Risk ratios (RRs) and hazard ratios (HRs) with 95% confidence intervals (CIs) were calculated for each outcome. The greedy nearest-neighbor algorithm with a caliper of 0.1 pooled SDs was used for matching. Continuous variables are represented as mean ± SD and were compared between the groups using independent-sample Student’s *t*-tests. Categorical variables are reported as count (percentage) and were compared between the groups using the chi-square test. A two-sided *p* value less than 0.05 was considered statistically significant. All statistical analyses were performed on the TriNetX network.

## 3. Results

We identified 876 patients with CPFE with antifibrotic use (cohort 1) and 4161 patients with CPFE who were not on antifibrotics (cohort 2). The baseline mean age +/− SD at diagnosis was 77.2 +/− 8.6 for patients with CPFE on antifibrotics and 77.9 +/− 10.5 for patients with CPFE who were not on antifibrotics. Some demographics differed in both cohorts, including race (white, 84% vs. 76%, respectively) and gender (male, 68% vs. 56%, respectively). The prevalence of comorbidities also differed between the two groups. Compared to those not on antifibrotics, CPFE patients on antifibrotics had a higher likelihood of having bronchiectasis (29%) and pulmonary hypertension (24%) and a lower likelihood of asthma (13.5%), chronic kidney disease (9.9%), and heart failure (29.7%). Furthermore, CPFE patients on antifibrotics were less likely to be on mycophenolate mofetil (7.8%) and more likely to be on bronchodilator therapy (anticholinergic 65.9%, sympathomimetic 84.5%, anti-inflammatory 60.8%), anti-lipemics (63.0%), and prednisone (61.5%).

After propensity score matching, 861 patients were identified in both cohorts. Baseline demographics were similar between the groups, including race (approximately 84% white) and gender (approximately 68% male). Other parameters, such as comorbidities and medication usage, were also not statistically different between the groups. Notable laboratory values showed an elevated hemoglobin A1c (approximately 6.3%), brain natriuretic peptide (approximately 1500 pg/mL), C-reactive protein (25 mg/L), and BMI (approximately 28 kg/m^2^) but were not statistically different between the groups.

Regarding the primary outcomes, the five-year follow-up after the index event showed a trend towards a higher risk of mortality in CPFE patients with antifibrotic use compared to patients without antifibrotic outcomes (HR: 1.14; CI 0.99–1.33; *p* 0.06), as listed in Table 2. No difference was found in the one-year follow-up (HR: 1.00; CI 0.84–1.20; *p* 0.93) in CPFE patients with antifibrotic use compared to those without, as shown in Table 3. However, at three-year follow-up (HR: 1.30; CI 1.11–1.53; *p* < 0.01), there was a statistically significant increased risk of mortality in CPFE patients with antifibrotic use compared to those without, as seen in Table 4.

Secondary outcomes included incidence of MACE (including MI and unstable angina), acute hypoxic respiratory failure, acute hypercapnic respiratory failure, and stroke. The five-year follow-up showed a trend toward increased risk of acute hypoxic respiratory failure (HR: 1.17; CI 0.99–1.39; *p* 0.06) and MI (HR: 1.68; CI 0.88–3.18; *p* 0.10). It also showed a trend towards a lower risk of stroke (HR 0.73; CI 0.51–1.05; *p* 0.86) and no differences for unstable angina (HR 0.94; CI 0.47–1.86; *p* 0.86) or acute hypercapnic respiratory failure (HR 0.99; CI 0.67–1.47; *p* 0.32). Similar trends were also noted in the one-year and three-year follow-ups, which have been included in Table 3 and Table 4.

## 4. Discussion

To the best of our knowledge, this is the first study examining the utilization of antifibrotic therapy for patients with CPFE over a 5-year follow-up period. Our results showed that patients on antifibrotics had a higher risk of mortality and complications such as MI or acute hypoxic respiratory failure. There was less risk of stroke and no difference in acute hypercapnic respiratory failure or unstable angina; however, these results might be potentially confounded by the “sicker patient” bias and would require prospective studies with well-defined cohorts in the future to appropriately answer this question.

Our findings are relevant, since there are no current evidence-based guidelines for targeted pharmacotherapy for CPFE. At this time, treatment for CPFE is based on supportive therapy or management of comorbidities such as tobacco use, pulmonary hypertension, chronic respiratory failure, and cardiovascular disease. However, this is based on an individualized approach, which leads to treatment of each patient separately due to a lack of uniformity [12].

Initial supportive therapies such as oxygen supplementation, smoking cessation [8], and pulmonary rehabilitation [13] have been proposed. Another approach to treatment includes targeting each component of CPFE, with specific and separate treatments for PF and emphysema. For the emphysema component, previous studies showed that CPFE patients who were started on inhaled bronchodilators and inhaled corticosteroids showed improvement in lung function compared to those who were not [14]. This is reflected in our study, as our patient population was already on bronchodilator therapy at baseline.

Treating the PF component requires more nuance. The landmark trials for antifibrotics have shown benefits for PF but do not specifically include CPFE. The ASCEND and CAPACITY trials for pirfenidone did show a benefit in survival for PF patients; however, patients with obstructive physiology (defined as FEV1/FVC < 0.8) were excluded from the study [10,15]. The INPULSIS trial for nintentanib showed a significant slowing of progression in patients with PF, although patients with obstructive physiology (defined as FEV1/FVC < 0.7) were also excluded [9]. Furthermore, the INBUILD trial for nintentanib went a step further and showed a reduction in disease progression for a range of interstitial lung diseases other than PF [16]. All of these trials also excluded patients with pre-existing comorbidities such as cardiovascular disease or renal disease. This differs from the patient population in our study, as our patients had cardiovascular/renal disease at baseline, reflected by the use of multiple cardiovascular medications and elevated brain natriuretic peptide at baseline.

Prior studies comparing outcomes between CPFE and isolated PF have been conflicting. In patients with CPFE, some studies have shown increased mortality [5,17], while others have shown a decreased mortality [18] when compared to isolated PF. Some speculate that there may be a survival benefit in the emphysematous component in CPFE due to the preservation of lung volumes [19].

Interestingly, our study showed that CPFE patients who were using antifibrotics actually had a trend towards higher risks of mortality, MI, and acute hypoxic respiratory failure at the five year follow-up. There was also noted a trend towards a lower risk of stroke and no difference in unstable angina or acute hypercapnic respiratory failure; however, these were likely hypothesis-generating signals due to the lack of statistical significance at the five-year follow-up. Notably, at the three-year follow-up, there was a statistically significant increased risk of mortality for CPFE patients with antifibrotic use compared to those without. This was surprising, given the promising benefits of antifibrotics on PF. It is important to interpret these findings with caution, given the concern for confounding by indication.

This result of increased risk of mortality in our CPFE cohort on antifibrotics must be considered an observation, which is overwhelmingly likely to be driven by uncontrolled confounding by indication (i.e., sicker patients receiving the drug). This can be evidenced by our study population being prescribed steroid, biological, and immunosuppressant therapy before the use of antifibrotics. However, given the exclusion of CPFE from pivotal antifibrotic trials, this finding raises the hypothesis that the known benefits of antifibrotics in pure IPF may not translate favorably to the CPFE phenotype. This observation is not conclusive of harm but strongly suggests that dedicated prospective studies or large, highly validated CPFE-specific registries are critically needed to determine the true efficacy and safety profile of antifibrotics in this high-risk population.

This study has several limitations. The primary limitation is the reliance of ICD-10 codes for the diagnosis of CPFE via the TriNetX database. This can potentially lead to inaccurate classification of CPFE, as the diagnosis mainly is reliant upon physiological testing and HRCT imaging. However, this approach was necessary to utilize the generalizability of the TriNetX database. Our study used a narrow set of ICD-10 codes that have been validated for specific diseases such as IPF. Studies have shown that using these codes had a positive predictive value of up to 75%, which increased up to 80% when confirmed by diagnostic testing [20]. Although imperfect, this approach could be used for case identification. However, it should be noted that there is still chance of misclassification with the diagnosis of CPFE.

Furthermore, important information such as medication dosing and compliance, pulmonary function spirometry (such as functional vital capacity and diffusing capacity of the lung for carbon monoxide), radiological severity (such as extent of fibrosis and emphysema from high-resolution computed tomography imaging), and severity of comorbidities could not be obtained throughout the study period. The inability to account for these variables can serve as potential confounding variables, which may affect the observed results of this study. Even after propensity score matching, these unaccounted factors could suggest that patients on antifibrotic therapy may represent a cohort with more severe and progressive disease. This could bias the results towards the null hypothesis or even suggest worse outcomes for the treatment group. Moreover, this could suggest the impact of antifibrotic medications instead of the effects of these medications on CPFE. Lastly, this retrospective study, especially with these limitations, cannot dictate causality and can only determine correlations.

## 5. Conclusions

In conclusion, in a real-world, clinically coded cohort of patients with CPFE, those on antifibrotics demonstrated increased rates of mortality, MACE, and respiratory failure. These results were likely driven by confounding by indication, with treatment of patients with more severe and progressive disease. Further studies with randomized controlled trials or further registry data should be performed to accurately assess the effects of antifibrotics on patients with CPFE.

## Figures and Tables

**Table 1:** Baseline characteristics before and after propensity score matching for cohorts 1 and 2.

	Before Propensity Score Matching			After Propensity Score Matching		
Characteristics	Cohort 1 (N = 876)	Cohort 2 (N = 4161)	*p* Value	Cohort 1 (N = 861)	Cohort 2 (N = 861)	*p* Value
Demographics						
Current Age	77.2 +/− 8.6	77.9 +/− 10.5	0.083	77.3 +/− 8.6	77.4 +/− 10.3	0.699
Age at Index	70.7 +/− 9.1	71.5 +/− 11.4	0.056	70.8 +/− 9.1	70.8 +/− 10.9	0.924
White	84.0%	76.5%	<0.001	84.1%	84.7%	0.740
Male	68.9%	56.2%	<0.001	68.5%	67.7%	0.717
Female	31.1%	43.8%	<0.001	31.5%	32.3%	0.717
Hispanic of Latino	6.5%	8.1%	0.116	6.4%	5.9%	0.688
Not Hispanic or Latino	81.7%	74.5%	<0.001	81.8%	80.7%	0.578
Black or African American	6.1%	11.8%	<0.001	6.2%	5.3%	0.469
Asian	2.7%	2.6%	0.872	2.6%	2.0%	0.418
Diagnoses						
Asthma	13.5%	18.2%	0.001	13.7%	14.4%	0.677
Bronchiectasis, Uncomplicated	29.1%	19.6%	<0.001	28.5%	28.1%	0.872
Hypertensive Chronic Kidney Disease	9.9%	15.1%	<0.001	10.1%	8.5%	0.245
Hypertensive Heart Disease	13.4%	13.4%	0.951	13.4%	12.1%	0.426
Essential (Primary) Hypertension	70.5%	69.8%	0.657	70.0%	70.6%	0.792
Chronic Ischemic Heart Disease	52.5%	47.1%	0.003	52.4%	50.1%	0.335
Heart Failure	29.7%	35.7%	0.001	30.0%	28.0%	0.367
Diabetes Mellitus	29.1%	31.1%	0.235	28.8%	27.2%	0.452
Overweight, Obesity, And other Hyperalimentation	29.3%	25.1%	0.009	29.0%	26.0%	0.161
Hyperlipidemia, Unspecified	56.6%	54.0%	0.150	56.0%	56.0%	1
Neoplasms	39.4%	40.6%	0.516	39.5%	39.5%	1
Obstructive Sleep Apnea (Adult) (Pediatric)	28.9%	23.4%	0.001	28.6%	28.1%	0.831
Pulmonary Hypertension, Unspecified	24.0%	16.3%	<0.001	23.2%	22.4%	0.688
Cerebral Infarction	5.6%	6.5%	0.299	5.7%	4.8%	0.386
Nontraumatic Intracerebral Hemorrhage	1.1%	0.5%	0.015	1.2%	1.2%	1
Medications						
Immunosuppressants						
Mycophenolate mofetil	7.8%	11.4%	0.002	7.8%	8.0%	0.858
Tacrolimus	6.2%	8.2%	0.038	6.0%	5.6%	0.680
Azathioprine	5.6%	6.5%	0.323	5.5%	5.6%	0.916
Basiliximab	3.1%	3.2%	0.861	3.0%	3.3%	0.782
Mycophenolic Acid	1.7%	3.1%	0.027	1.7%	2.1%	0.598
Cyclosporine	1.9%	2.3%	0.560	1.9%	2.1%	0.729
Sirolimus	1.1%	1.3%	0.629	1.2%	0%	0.002
Infliximab	1.1%	0.5%	0.028	1.2%	1.2%	1
Ustekinumab	0%	0.2%	0.146	0%	0%	-
Imiquimod	1.1%	0.4%	0.010	1.2%	1.2%	1
Biological Therapy						
Etanercept	1.1%	0.7%	0.172	1.2%	1.2%	1
Adalimumab	1.1%	0.6%	0.121	1.2%	1.2%	1
Inhaled Therapies						
Anti-Inflammatories	60.8%	51.2%	<0.001	60.7%	60.2%	0.805
Bronchodilators, Anticholinergic	65.9%	59.4%	<0.001	65.7%	65.0%	0.761
Bronchodilators, Sympathomimetic	84.5%	75.9%	<0.001	84.2%	82.9%	0.474
Vilanterol	14.5%	9.8%	<0.001	14.4%	13.8%	0.729
Cromolyn	1.1%	0.2%	<0.001	1.2%	1.2%	1
Montelukast	15.3%	11.6%	0.002	15.3%	16.1%	0.643
Miscellaneous						
Anti-Neoplastics	9.7%	11.1%	0.219	9.6%	8.6%	0.451
Cardiovascular						
Anti-Lipemics	63.0%	55.0%	<0.001	62.5%	60.4%	0.373
Antiarrhythmics	60.8%	58.8%	0.265	60.9%	59.8%	0.658
Diuretics	53.5%	55.4%	0.309	53.7%	50.9%	0.247
Beta-Blockers	53.8%	55.3%	0.400	54.1%	53.1%	0.664
Calcium Channel Blockers	36.0%	38.5%	0.163	36.1%	32.6%	0.128
Angiotensin-Converting Enzyme (ACEI) Inhibitors	23.7%	25.3%	0.332	24.0%	22.4%	0.424
Angiotensin II Inhibitors	23.5%	20.0%	0.018	22.9%	23.1%	0.909
Corticosteroids						
Prednisone	61.5%	55.8%	0.002	61.4%	61.3%	0.961
Methylprednisolone	43.4%	41.0%	0.189	43.4%	44.8%	0.560
Dexamethasone	25.6%	22.7%	0.064	25.4%	26.9%	0.476
Triamcinolone	16.4%	17.2%	0.606	16.4%	16.3%	0.948
Hydrocortisone	12.3%	14.8%	0.060	12.4%	13.2%	0.614
Prednisolone	5.0%	5.0%	1	5.0%	4.9%	0.911
Betamethasone	6.6%	6.5%	0.927	6.7%	6.7%	1

Brain Natriuretic Peptide (serum)	1465.4 +/− 2705.1	2663.2 +/− 6529.3	0.014	1473.6 +/− 2717.7	1507.6 +/− 2754.0	0.905
Hemoglobin A1c in Blood	6.1 +/− 1.0	6.3 +/− 1.2	0.009	6.1 +/− 1.0	6.3 +/− 1.3	0.124
C-Reactive Protein (Serum)	23.0 +/− 45.9	29.3 +/− 49.5	0.059	23.5 +/− 46.6	29.6 +/− 55.5	0.185
BMI	28.3 +/− 5.8	27.8 +/− 6.5	0.054	28.3 +/− 5.8	27.9 +/− 6.2	0.173

**Table 2:** Effects of antifibrotic therapy on CPFE patients at 5-year follow-up.

5-Year Follow-Up		Cohort 1 (Using Antifibrotics)	Cohort 2 (Not Using Antifibrotics)	
Outcomes ^a^		5-Year Follow-Up	5-Year Follow-Up	RR * or HR * (95% CI) *p* Value **
**Overall Mortality**	**n after matching ^b^**	856	851	HR 1.14 CI (0.99, 1.33) *p*: 0.06
	**Patients with outcome**	377	332	
**Incidence of acute hypoxic respiratory failure**	**n after matching ^b^**	861	861	HR 1.17 CI (0.99, 1.39) *p*: 0.06
	**Patients with outcome**	279	246	
**Incidence of acute hypercapnic respiratory failure**	**n after matching ^b^**	861	861	HR 0.99 CI (0.67, 1.47) *p*: 0.97
	**Patients with outcome**	50	50	
**Incidence of myocardial infarction**	**n after matching ^b^**	861	861	HR 1.68 CI (0.88, 3.18) *p*: 0.10
	**Patients with outcome**	25	15	
**Incidence of stroke**	**n after matching ^b^**	861	861	HR 0.73 CI (0.51, 1.05) *p*: 0.08
	**Patients with outcome**	53	71	
**Incidence of unstable angina**	**n after matching ^b^**	861	861	HR 0.94 CI (0.47, 1.86) *p*: 0.86
	**Patients with outcome**	16	17	

* RR: risk ratio; HR: hazard ratio. ** 95% CI, 95% confidence interval, Long-rank Test *p* value. ^a^ Propensity matching balanced cohorts according to demographic variables, laboratory results, associated comorbidities, and use of medications at baseline. ^b^ Patients who had the outcome before the time window were excluded from each cohort.

**Table 3:** Effects of antifibrotic therapy on CPFE patients at 3-year follow-up.

3-Year Follow-Up		Cohort 1 (Using Antifibrotics)	Cohort 2 (Not Using Antifibrotics)	
Outcomes ^a^		3-Year Follow-Up	3-Year Follow-Up	RR * or HR * (95% CI) *p* Value **
**Overall Mortality**	**n after matching ^b^**	819	817	HR 1.30 CI (1.11, 1.53) *p*: <0.01
	**Patients with outcome**	362	277	
**Incidence of acute hypoxic respiratory failure**	**n after matching ^b^**	823	823	HR 0.97 CI (0.81, 1.17) *p*: 0.82
	**Patients with outcome**	222	224	
**Incidence of acute hypercapnic respiratory failure**	**n after matching ^b^**	823	823	HR 0.81 CI (0.54, 1.22) *p*: 0.32
	**Patients with outcome**	44	52	
**Incidence of myocardial infarction**	**n after matching ^b^**	823	823	HR 1.20 CI (0.64, 2.25) *p*: 0.55
	**Patients with outcome**	22	18	
**Incidence of stroke**	**n after matching ^b^**	823	823	HR 0.90 CI (0.61, 1.33) *p*: 0.62
	**Patients with outcome**	49	53	
**Incidence of unstable angina**	**n after matching ^b^**	823	823	HR 0.93 CI (0.46, 1.88) *p*: 0.84
	**Patients with outcome**	15	16	

* RR: risk ratio; HR: hazard ratio. ** 95% CI, 95% confidence interval, Long-rank Test *p* value. ^a^ Propensity matching balanced cohorts according to demographic variables, laboratory results, associated comorbidities, and use of medications at baseline. ^b^ Patients who had the outcome before the time window were excluded from each cohort.

**Table 4:** Effects of antifibrotic therapy on CPFE patients at 1-year follow-up.

1-Year Follow Up		Cohort 1 (Using Antifibrotics)	Cohort 2 (Not Using Antifibrotics)	
Outcomes ^a^		1-Year Follow-Up	1-Year Follow-Up	RR * or HR * (95% CI) *p* Value **
**Overall Mortality**	**n after matching ^b^**	835	833	HR 1.00 CI (0.84, 1.20) *p*: 0.93
	**Patients with outcome**	244	234	
**Incidence of acute hypoxic respiratory failure**	**n after matching ^b^**	840	840	HR 0.99 CI (0.79, 1.24) *p*: 0.93
	**Patients with outcome**	150	150	
**Incidence of acute hypercapnic respiratory failure**	**n after matching ^b^**	840	840	HR 0.78 CI (0.46, 1.32) *p*: 0.36
	**Patients with outcome**	25	31	
**Incidence of myocardial infarction**	**n after matching ^b^**	840	840	HR 1.17 CI (0.59, 2.33) *p*: 0.63
	**Patients with outcome**	18	15	
**Incidence of stroke**	**n after matching ^b^**	840	840	HR 0.76 CI (0.48, 1.20) *p*: 0.24
	**Patients with outcome**	33	42	
**Incidence of unstable angina**	**n after matching ^b^**	840	840	HR 2.67 CI (0.85, 8.41) *p*: 0.07
	**Patients with outcome**	11	10	

* RR: risk ratio; HR: hazard ratio. ** 95% CI, 95% confidence interval, Long-rank Test *p* value. ^a^ Propensity matching balanced cohorts according to demographic variables, laboratory results, associated comorbidities, and use of medications at baseline. ^b^ Patients who had the outcome before the time window were excluded from each cohort.

## Data Availability

The data that support the findings of this study are available from TrinetX. Restrictions apply to the availability of these data, which were used under license for this study. Data are available at URL https://trinetx.com with the permission of TrinetX.

## Abbreviations

The following abbreviations are used in this manuscript:

CPFE	Chronic Pulmonary Fibrosis and Emphysema
PF	Pulmonary Fibrosis
HRCT	High-Resolution Computed Tomography
MACE	Major Adverse Cardiac Event
MI	Myocardial Infarction
RR	Relative Risk
HR	Hazard Ratio
CT	Computed Tomography
CI	Confidence Interval
BMI	Body Mass Index
FEV1	Forced Expiratory Volume
FVC	Forced Vital Capacity
CRP	C-Reactive Protein
ACEI	Angiotensin-Converting Enzyme
PSM	Propensity Score Matching

## References

[1] Cottin V., Nunes H., Brillet P.Y., Delaval P., Devouassaoux G., Tillie-Leblond I., Israel-Biet D., Court-Fortune I., Valeyre D., Cordier J.-F. (2005). Combined pulmonary fibrosis and emphysema: A distinct underrecognised entity. Eur. Respir. J..

[2] Jankowich M.D., Rounds S.I.S. (2012). Combined Pulmonary Fibrosis and Emphysema Syndrome: A Review. Chest.

[3] Hage R., Gautschi F., Steinack C., Schuurmans M.M. (2021). Combined Pulmonary Fibrosis and Emphysema (CPFE) Clinical Features and Management. Int. J. Chron. Obstruct. Pulmon. Dis..

[4] Lee C.H., Kim H.J., Park C.M., Lim K.Y., Lee J.Y., Kim D.J., Yeon J.H., Hwang S.-S., Kim D.-K., Lee S.-M. (2011). The impact of combined pulmonary fibrosis and emphysema on mortality. Int. J. Tuberc. Lung Dis..

[5] Sugino K., Ishida F., Kikuchi N., Hirota N., Sano G., Sato K., Isobe K., Sakamoto S., Takai Y., Homma S. (2014). Comparison of clinical characteristics and prognostic factors of combined pulmonary fibrosis and emphysema versus idiopathic pulmonary fibrosis alone. Respirology.

[6] Cottin V., Le Pavec J., Prévot G., Mal H., Humbert M., Simonneau G., Cordier J.-F. (2009). Pulmonary hypertension in patients with combined pulmonary fibrosis and emphysema syndrome. Eur. Respir. J..

[7] Koo H.J., Do K.H., Lee J.B., Alblushi S., Lee S.M. (2016). Lung Cancer in Combined Pulmonary Fibrosis and Emphysema: A Systematic Review and Meta-Analysis. PLoS ONE.

[8] Cottin V., Inoue Y., Selman M., Ryerson C.J., Wells A.U., Agusti A., Wong A.W., Corte T.J., Flaherty K.R., Han M.K. (2022). Syndrome of Combined Pulmonary Fibrosis and Emphysema: An official research statement from American Thoracic Society (ATS), European Respiratory Society (ERS), Japanese Respiratory Society (JRS), and Asociación Latinoamericana de Tórax (ALAT). Am. J. Respir. Crit. Care Med..

[9] Richeldi L., du Bois R.M., Raghu G., Azuma A., Brown K.K., Costabel U., Cottin V., Flaherty K.R., Hansell D.M., Inoue Y. (2014). Correction: Efficacy and Safety of Nintedanib in Idiopathic Pulmonary Fibrosis. N. Engl. J. Med..

[10] King T.E., Bradford W.Z., Castro-Bernardini S., Fagan E.A., Glaspole I., Glassberg M.K., Gorina E., Hopkins P.M., Kardatzke D., Lancaster L. (2014). A Phase 3 Trial of Pirfenidone in Patients with Idiopathic Pulmonary Fibrosis. N. Engl. J. Med..

[11] Palchuk M.B., London J.W., Perez-Rey D., Drebert Z.J., Winer-Jones J.P., Thompson C.N., Esposito J., Claerhout B. (2023). A global federated real-world data and analytics platform for research. JAMIA Open.

[12] Wong A.W., Liang J., Cottin V., Ryerson C.J. (2020). Diagnostic Features in Combined Pulmonary Fibrosis and Emphysema: A Systematic Review. Ann. Am. Thorac. Soc..

[13] Tomioka H., Mamesaya N., Yamashita S., Kida Y., Kaneko M., Sakai H. (2016). Combined pulmonary fibrosis and emphysema: Effect of pulmonary rehabilitation in comparison with chronic obstructive pulmonary disease. BMJ Open Respir. Res..

[14] Dong F., Zhang Y., Chi F., Song Q., Zhang L., Wang Y., Che C. (2015). Clinical efficacy and safety of ICS/LABA in patients with combined idiopathic pulmonary fibrosis and emphysema. Int. J. Clin. Exp. Med..

[15] Noble P.W., Albera C., Bradford W.Z., Costabel U., Glassberg M.K., Kardatzke D., King T.E., Lancaster L., Sahn S.A., Szwarcberg J. (2011). Pirfenidone in patients with idiopathic pulmonary fibrosis (CAPACITY): Two randomised trials. Lancet.

[16] Flaherty K.R., Wells A.U., Cottin V., Devaraj A., Walsh S.L.F., Inoue Y., Richeldi L., Kolb M., Tetzlaff K., Stowasser S. (2019). Nintedanib in Progressive Fibrosing Interstitial Lung Diseases. N. Engl. J. Med..

[17] Mejía M., Carrillo G., Rojas-Serrano J., Estrada A., Suárez T., Alonso D., Barrientos E., Gaxiola M., Navarro C., Selman M. (2009). Idiopathic pulmonary fibrosis and emphysema: Decreased survival associated with severe pulmonary arterial hypertension. Chest.

[18] Kurashima K., Takayanagi N., Tsuchiya N., Kanauchi T., Ueda M., Hoshi T., Miyahara Y., Sugita Y. (2010). The effect of emphysema on lung function and survival in patients with idiopathic pulmonary fibrosis. Respirology.

[19] Cottin V., Hansell D.M., Sverzellati N., Weycker D., Antoniou K.M., Atwood M., Oster G., Kirchgaessler K.-U., Collard H.R., Wells A.U. (2017). Effect of emphysema extent on serial lung function in patients with idiopathic pulmonary fibrosis. Am. J. Respir. Crit. Care Med..

[20] Morgan A., Gupta R.S., George P.M., Quint J.K. (2023). Validation of the recording of idiopathic pulmonary fibrosis in routinely collected electronic healthcare records in England. BMC Pulm. Med..

